# Bortezomib augments lymphocyte stimulatory cytokine signaling in the tumor microenvironment to sustain CD8^+^T cell antitumor function

**DOI:** 10.18632/oncotarget.14365

**Published:** 2016-12-29

**Authors:** Samuel T. Pellom, Duafalia F. Dudimah, Menaka C. Thounaojam, Roman V. Uzhachenko, Ashutosh Singhal, Ann Richmond, Anil Shanker

**Affiliations:** ^1^ Department of Biochemistry and Cancer Biology, School of Medicine, Meharry Medical College, Nashville, Tennessee, USA; ^2^ Department of Microbiology and Immunology, School of Medicine, Meharry Medical College, Nashville, Tennessee, USA; ^3^ School of Graduate Studies and Research, Meharry Medical College, Nashville, Tennessee, USA; ^4^ Tennessee Valley Healthcare System, Nashville, Tennessee, USA; ^5^ Department of Veterans Affairs, Nashville, Tennessee, USA; ^6^ Department of Cancer Biology, Vanderbilt University Medical Center, Nashville, Tennessee, USA; ^7^ Host-Tumor Interactions Research Program, Vanderbilt-Ingram Comprehensive Cancer Center, Vanderbilt University, Nashville, Tennessee, USA; ^8^ Vanderbilt Center for Immunobiology, Vanderbilt University, Nashville, Tennessee, USA; ^9^ Vanderbilt Center for Translational and Clinical Immunology, Vanderbilt University, Nashville, Tennessee, USA

**Keywords:** proteasome inhibition, CD8^+^ T cells, immunosuppression, cancer immunotherapy, adoptive cell therapy

## Abstract

Tumor-induced immune tolerance poses a major challenge for therapeutic interventions aimed to manage cancer. We explored approaches to overcome T-cell suppression in murine breast and kidney adenocarcinomas, and lung fibrosarcoma expressing immunogenic antigens. We observed that treatment with a reversible proteasome inhibitor bortezomib (1 mg/kg body weight) in tumor-bearing mice significantly enhanced the expression of lymphocyte-stimulatory cytokines IL-2, IL-12, and IL-15. Notably, bortezomib administration reduced pulmonary nodules of mammary adenocarcinoma 4T1.2 expressing hemagglutinin (HA) model antigen (4T1HA) in mice. Neutralization of IL-12 and IL-15 cytokines with a regimen of blocking antibodies pre- and post-adoptive transfer of low-avidity HA_518-526_-specific CD8^+^T-cells following intravenous injection of 4T1HA cells increased the number of pulmonary tumor nodules. This neutralization effect was counteracted by the tumor metastasis-suppressing action of bortezomib treatments. In bortezomib-treated 4T1HA tumor-bearing mice, CD4^+^T-cells showed increased IL-2 production, CD11c^+^ dendritic cells showed increased IL-12 and IL-15 production, and HA-specific activated CD8^+^T-cells showed enhanced expression of IFNγ, granzyme-B and transcription factor eomesodermin. We also noted a trend of increased expression of IL-2, IL-12 and IL-15 receptors as well as increased phosphorylation of STAT5 in tumor-infiltrating CD8^+^T-cells following bortezomib treatment. Furthermore, bortezomib-treated CD8^+^T-cells showed increased phosphorylation of mitogen-activated protein kinase p38, and Akt, which was abrogated by phosphatidylinositide 3-kinase (PI3K) inhibitor. These data support the therapeutic potential of bortezomib in conjunction with other immunotherapies to augment the strength of convergent signals from CD8^+^T-cell signaling molecules including TCR, cytokine receptors and downstream PI3K/Akt/STAT5 pathways to sustain CD8^+^T-cell effector function in the tumor microenvironment.

## INTRODUCTION

The immunosuppressive tumor microenvironment usurps host anti-tumor immune responses making cancer treatment extremely complex and challenging. Therapeutic approaches that subvert tumor-induced immune suppression and control tumor metastasis are highly sought after in a hope to provide relapse-free survival in cancer patients. A predominance of immune stimulatory cytokines over a suppressive milieu in the tumor microenvironment decides the outcome of therapeutic protocols. Tumors negatively impact effector functions of immune cells through immunosuppressive molecules, such as PD-L1 (B7-H1) and soluble factors, such as transforming growth factor beta (TGFβ), interleukin-10 (IL-10) and vascular endothelial growth factor (VEGF) [1]. Among multiple tumor-associated suppressive mechanisms, tumors also downregulate the evolutionarily conserved Notch signaling, crucial in several biological processes including immune cell differentiation and effector function [2, 3]. Evidence is emerging to support that a functional crosstalk between Notch and nuclear factor-κB (NFκB) signaling pathways are necessary for antitumor CD8^+^T cell effector and memory differentiation [4–7].

Immunostimulatory cytokines (e.g., IL-2, IL-12, IL-15) support lymphocyte differentiation and survival enabling tumor control [3]. Various approaches to overcome tumor-induced immunosuppression using exogenous cytokines as well as immune adjuvants and modulators, dendritic cell vaccines, and adoptive immune cell transfers have not been successful clinically, albeit promising initial results. Bortezomib (Velcade^TM^/PS-341) is a FDA-approved drug for the treatment of multiple myeloma and mantle cell lymphoma [8–14]. It is a dipeptidyl boronate that inhibits the ubiquitin-proteasome proteolytic pathway. The boronic acid moiety of bortezomib with its transient (half-life of 9-15 h) and reversible inhibition of the proteasome set it apart from other proteasome inhibitors as a therapeutic agent. Unlike other non-boronic acid proteasome inhibitors, the boronic acid moiety of bortezomib prevents it from being transported out of the cell by the multidrug resistance system [15]. It has been observed that bortezomib administration enhances the expression of TNF-R1 and IFN-γ-Rα in TBJ neuroblastoma cells [16] suggesting that bortezomib may potentially modulate the tumor microenvironment. In our previous studies, we showed that bortezomib can also sensitize various mouse and human solid tumor cells to apoptosis by tumor necrosis factor family members [17, 18]. Although there are reports of negative side effects of bortezomib on some immune cells, such as a reduction in dendritic cell and leukocyte numbers [19–24], studies have lately suggested that bortezomib, directly or indirectly, may enhance immune functions under optimized conditions [25–32]. We also observed that treatment with bortezomib was associated with an increased crosstalk between Notch and NFκB signaling pathways in effector T cells [7], and an improved response to adoptive T cell therapy by enhanced FasL cytotoxicity [33]. However, it is not well understood how bortezomib affects the cytokine milieu in the tumor microenvironment that could influence antitumor T cell immunity and tumor burden.

In this study, using a therapeutic regimen established by us previously [17], we explored the effects of bortezomib administered at a dose of 1 mg/kg body weight on the cytokine milieu and CD8^+^T cell function in mice bearing murine mammary and renal adenocarcinomas that present a defined low-avidity MHC-I-restricted epitope (IYSTVASSL) derived from a hemagglutinin (HA) model antigen, or lung fibrosarcoma that expresses human Ras and mutant p53 as xenogenic antigens. We show that bortezomib administration in tumor-bearing mice modulates the levels of immunostimulatory cytokines IL-2, IL-12, and IL-15 and enhances their downstream signaling by increasing the expression of their receptors on tumor-infiltrating CD8^+^T cells in endogenous or adoptive HA_518-526_-specific CD8^+^T cell transfer set ups resulting in reduced pulmonary metastatic nodules. Data also show that these effects of bortezomib treatment are mediated *via* the activation of PI3K/Akt/STAT5 pathways in CD8^+^T cells enhancing their effector function. These findings suggest that besides bortezomib's established role in sensitizing tumors to apoptosis, it also has immunostimulatory potential to therapeutically modulate the tumor microenvironment with a carefully optimized bortezomib regimen to sustain lymphocytic effector function, and overcome tumor-associated immunosuppression.

## RESULTS

### Bortezomib treatment affects the cytokine milieu in tumor-bearing mice

**Figure 1:**
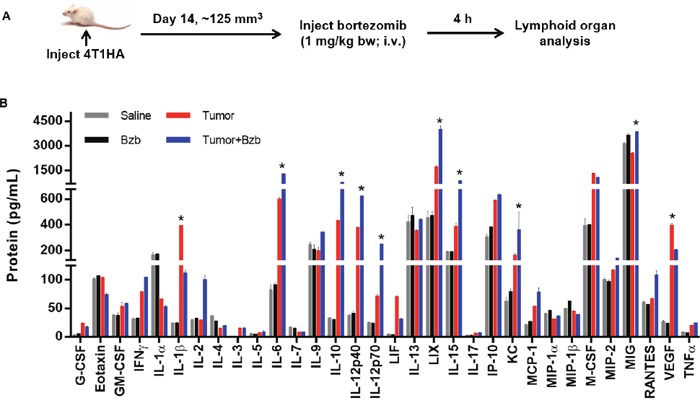
Modulation of cytokine/chemokine expression by bortezomib in 4T1HA tumor-bearing mice **A.** Orthotopic tumors were established in mammary pads of Balb/c wild type mice following injection of 2 × 106 4T1HA tumor cells. On day 14 (after the tumor reached at least a size of 125 mm3), mice were injected intravenously with 1 mg/kg body weight of bortezomib (Bzb, ~20 nM by total blood volume in 8-wk mouse). After 4 h, the mice were euthanized and total cell lysates were made from the RBC-depleted splenocytes and 245 μg of protein from these lysates were analyzed for cytokine/chemokine concentration by MagPix array (Millipore). **B.** MagPix output data constitutes protein concentration (pg/mL) of cytokines/chemokines modulated in the spleen of 4T1HA tumor-bearing mice. Four experimental groups were compared: Saline control (gray bar), naïve mice treated with Bzb alone (black bar), mice with tumor alone (red bar), and tumor-bearing mice treated with Bzb (blue bar). Protein concentration of analytes compared among the 4 groups is shown as means ± SD from 4 independent experiments. *p values are representative as *p<0.05 (ANOVA, one-way) and used to compare tumor alone to tumor+Bzb group.

**Figure 2:**
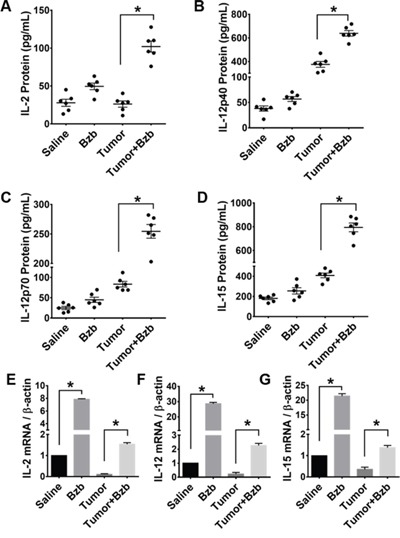
Effect of bortezomib administration on IL-2, IL-12, and IL-15 proteins and mRNA expression in vivo Protein expression of cytokines IL-2 **A.** IL-12p40 **B.** IL-12p70 **C.** IL-15 **D.** in total cell lysates of RBC-depleted splenocytes from Balb/c WT mice were quantified 4 h after Bzb (1 mg/kg body weight) administration by MagPix multiplex array. Cytokine mRNA expression levels measured by RT-PCR are shown as the ratio of target gene expression to housekeeping gene, β-actin, for IL-2 **E.** IL-12 **F.** IL-15 **G.** The bar graph is shown as mean ± SD from 2 independent experiments. *p < 0.05; (ANOVA, one-way).

**Figure 3:**
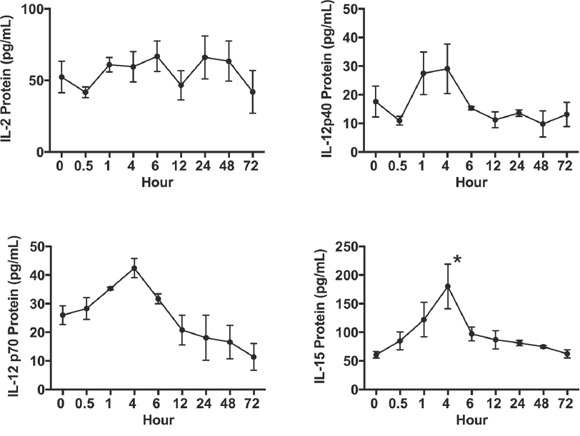
Time kinetics of splenic IL-2, IL-12 and IL-15 expression in vivo following bortezomib treatment Balb/c WT mice were administered intravenously with Bzb (1 mg/kg body weight) at the indicated time points over a 72 h period. Total cell lysate samples of RBC-depleted splenocytes were prepared from a group of mice at different time points. Protein levels for IL-2, IL-12p40, IL-12p70, and IL-15 were quantified using the MagPix multiplex array. Values are presented as mean ± SEM; ANOVA, one-way; *p<0.05 compared to other time points.

We investigated the effects of the reversible proteasome inhibitor drug bortezomib on the cytokine milieu in the tumor microenvironment of murine mammary 4T1.2 (representative of stage IV human breast cancer) [34] or Renca adenocarcinomas presenting a low-avidity HA_518-526_ epitope [35], or lung fibrosarcoma D459. In mice with large established (~125 mm^3^) orthotopic mammary tumors of 4T1HA cells, MagPix multiplex cytokine bead array showed that bortezomib treatment significantly increased protein levels of immunostimulatory cytokines IL-2, IL-12p40, IL-12p70, and IL-15, and decreased the levels of tumor-promoting cytokines IL-1β and VEGF in the splenic lysates when compared with protein levels in untreated mice with tumor alone (Figure 1 and Table 1). Significantly increased levels of IL-15 were observed in the serum of mice bearing 4T1HA as well as RencaHA or D459 tumors (Table 2). A similar trend of cytokine changes was observed in the lymph node (LN), tumor mass or thymus lysates from mice bearing 4T1HA, RencaHA or D459 tumors (data not shown). An increase in mRNA levels of IL-2, IL-12p40, IL-12p70, and IL-15 correlated with their increased protein levels in splenocytes of bortezomib-treated tumor-bearing mice compared with untreated tumor-bearing mice (Figure 2). Moreover, assessment of cytokine protein levels over the course of 72 h in naïve WT mice showed that expression of the immunostimulatory cytokines IL-2, IL-12p40, IL-12p70, and IL-15 (Figure 3) reached to their peak at 4 h after bortezomib administration.

**Table 1:** Expression of cytokines/chemokines in splenic lysates of 4T1HA tumor-bearing mice following bortezomib treatment

Protein (pg/mL)	Saline	Bzb	Tumor	Tumor+Bzb
**Immunostimulatory Cytokines**				
IL-2	27.9 ± 11.4	38.5 ± 13.9	26.2 ± 10.1	102.1 ± 17.8*
IL-12p40	38.0 ± 12.5	48.2 ± 15.7	375.8 ± 60.1	638.7 ± 58.4*
IL-12p70	27.0 ± 14.1	27.0 ± 10.2	83.4 ± 16.7	254.5 ± 28.5*
IL-15	180.2 ± 31.9	173.7 ± 43.0	408.1 ± 57.6	794.9 ± 90.3*
**Chemoattractants**				
MIG	3133.9 ± 126.9	3671.7 ± 134.4	2586 ± 30.4	3853 ± 44.0*
RANTES	59.9 ± 3.7	56.0 ± 1.7	66.6 ± 2.5	106.6 ± 9.0
MCP-1	21.1 ± 2.2	26.9 ± 1.7	54.0 ± 1.0	77.0 ± 10.4
MIP-1α	39.8 ± 2.7	46.3 ± 1.2	30.4 ± 0.4	35.9 ± 2.9
MIP-1β	49.6 ± 0.2	62.6 ± 1.2	44 ± 0.9	38.6 ± 0.3
MIP-2	100.1 ± 5.6	96.9 ± 3.4	117.1 ± 2.0	137.3 ± 0.4
**Effector Cytokines**				
IFNγ	31.5 ± 2.7	32.5 ± 0.8	78.6 ± 1.7	104.4 ± 0.2
TNFα	8.5 ± 0.1	6.4 ± 0.2	19.6 ± 0.4	23.7 ± 0.5
**Tumor-Promoting Cytokines/Factors**				
G-CSF	3.0 ± 0.5	5.6 ± 0.3	23.1 ± 1.7	17.2 ± 1.9
IL-1β	23.7 ± 1.1	24.1 ± 3.2	392.2 ± 3.3	111.5 ± 8.0*
M-CSF	388.4 ± 74.4	402.0 ± 4.6	1299.0 ± 40.3	1061.0 ± 45.5
VEGF	26.8 ± 2.4	23.1 ± 0.8	397.6 ± 21.4	201.5 ± 9.5*

4T1HA tumor cells (0.5 × 10^6^) were injected orthotopically in mammary pads to establish tumors approximately 125 mm^3^ in size. Bortezomib (1 mg/kg body weight) was administered intravenously in mice. Bortezomib (1 mg/kg body weight) was administered intravenously in mice, and protein array of various analytes in total splenic lysates was performed 4 h later by MagPix multiplex bead assay. Protein values are presented as mean ± SD. *p<0.05 (ANOVA, one-way), comparing Tumor vs Tumor+Bzb.

**Table 2:** Expression of cytokines in the serum of tumor-bearing mice following bortezomib treatment

Protein (pg/mL)	Saline	Bzb	Tumor	Tumor+Bzb
**Immunostimulatory Cytokines**:				
**4T1HA**				
IL-2	BDR	6.8 ± 1.1	BDR	3.4 ± 0.9
IL-12p40	12.5 ± 0.8	BDR	BDR	4.6 ± 1.6
IL-12p70	2.2 ± 0.7	BDR	BDR	BDR
IL-15	6.3 ± 4.9	11.1 ± 0.7	80.2 ± 25.9	313.4 ± 21.8*
**RencaHA**				
IL-2	BDR	19.5 ± 20.9	BDR	11.75 ± 15.2
IL-12p40	BDR	6.8 ± 8.2	16.2 ± 21.4	11.9 ± 1.6
IL-12p70	BDR	6.4 ± 7.6	BDR	15.2 ± 20.1
IL-15	BDR	33.9 ± 6.7*	27.1 ± 8.5	601.4 ± 19.5*
**D459**				
IL-2	5.7 ± 0	10.8 ± 2.6	9.9 ± 4.4	10.3 ± 1.4
IL-12p40	5.2 ± 1.1	9.9 ± 4.8	17 ± 5.8	17.3 ± 5.4
IL-12p70	10.3 ± 4.1	20 ± 4.5	23.7 ± 2.3	22.2 ± 2.3
IL-15	17.6 ± 9.6	38.7 ± 5.5*	38 ± 1.8	134.1 ± 3.9*

4T1HA cells (0.5 × 10^6^) were injected orthotopically and RencaHA or D459 cells subcutaneously to establish tumors approximately 125 mm^3^ in size. Bortezomib (1 mg/kg body weight) was administered intravenously in mice and protein array of immunostimulatory analytes in serum was performed 4 h later by MagPix multiplex bead assay. Protein values are presented as mean ± SD. *p<0.05 (ANOVA, one-way), comparing Tumor vs Tumor+Bzb. BDR: Below the minimum detection range (3.2 pg/mL).

These data suggest that bortezomib administration increases the expression of immunostimulatory cytokines IL-2, IL-12, and IL-15 at both the transcriptional and translational levels in tumor-bearing mice. The effects of bortezomib treatment on these cytokines, which are key players in the cytotoxic and memory response mediated by CD8^+^ T cells and NK cells [36–38], suggest that bortezomib has the potential to influence the tumor microenvironment and host antitumor immunity.

### Bortezomib treatment reduces tumor metastatic nodules in the lung

**Figure 4:**
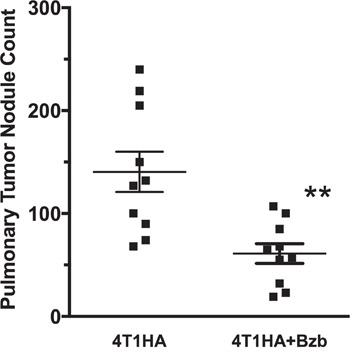
Effect of bortezomib on lung metastases of 4T1HA mammary tumor cells Balb/c wild type (WT) mice injected with 4T1HA tumor cells (0.5 × 106; i.v.; day 0) were administered intravenously with bortezomib (1 mg/kg body weight, ~20 nM by total blood volume) on days 4, 7, 11, and 15. Tumor metastases pulmonary nodules were counted on day 18. Nodule counts are presented as mean ± SEM; **p <0.01; two-tailed t-test.

**Figure 5:**
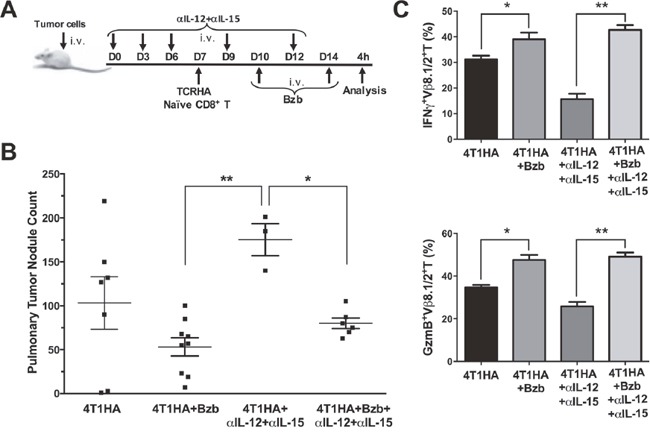
Bortezomib counteracts IL-12 and IL-15 neutralization by enhancing CD8+T cell effector molecules and reducing pulmonary nodules of 4T1HA tumor cells **A.** 0.5 × 106 4T1HA tumor cells were injected intravenously in Balb/c WT mice along with intravenous injections of IL-12 and IL-15 neutralizing antibodies on days 0, 3, 6, 9, and 12. On day 7, adoptive transfer of TCRHA CD8+T cells (2 × 106/mouse, i.v.) was performed. Bzb (1 mg/kg body weight) was administered on days 10 and 14. **B.** Lungs were harvested on day 14 to count tumor pulmonary nodules. Central lines depict values as means ± SEM. C. Bar graphs present data for IFNγ+Vb8.1/2+ and granzyme-B+Vβ8.1/2+ CD8+T cells. Values are depicted as means ± SD. *p<0.05, **p<0.01; (ANOVA, one-way).

Administration of a therapeutic regimen of bortezomib [17] given intravenously at 1 mg/kg body weight (~20 nM by blood volume) on days 4, 7, 11, and 15 after the intravenous injection of 4T1HA tumor cells in mice showed significant reduction in metastatic pulmonary nodules (Figure 4). To further understand bortezomib's effect on enhancing immune mechanisms and reducing tumor burden by modulating immunostimulatory cytokines, we investigated a therapeutic setup where we adoptively transferred Cln4 CD8^+^T cells specific to the HA_518-526_ epitope (Vβ8.1 clonotype) into 4T1HA tumor-bearing mice. In these mice, IL-12 and IL-15 cytokines were neutralized with a regimen of blocking antibodies pre- and post-adoptive transfer of HA_518-526_-specific CD8^+^T cells. Neutralization of IL-12 and IL-15 cytokines following intravenous injection of 4T1HA cells increased the number of tumor pulmonary nodules. This neutralization effect was counteracted by the tumor metastasis-suppressing action of bortezomib treatments (Figure 5A, 5B).

We also analyzed the expression of effector molecules on HA_518-526_-specific CD8^+^T cells in these mice following bortezomib treatments. We observed that IFNγ and granzyme-B expression on Vβ8.1/2^+^CD8^+^T cells in the spleens of tumor-bearing mice injected with IL-12 and IL-15 neutralization antibodies was significantly reduced compared with the expression in unneutralized tumor-bearing mice. Bortezomib administration increased IFNγ and granzyme-B expression on Vβ8.1/2^+^CD8^+^T cells in tumor-bearing mice even in the presence of IL-12 and IL-15 neutralization antibody treatments, and restored them to the levels comparable to tumor-bearing mice treated with bortezomib only (Figure 5C). These results suggest bortezomib administration overcomes IL-12 and IL-15 deficiency by increasing the expression of effector molecules and, thus, aids in reducing tumor pulmonary metastasis in 4T1HA-bearing mice.

### Bortezomib enhances production of IL-2 from CD4^+^T cells, and IL-12 and IL-15 from CD11c^+^ cells

**Figure 6:**
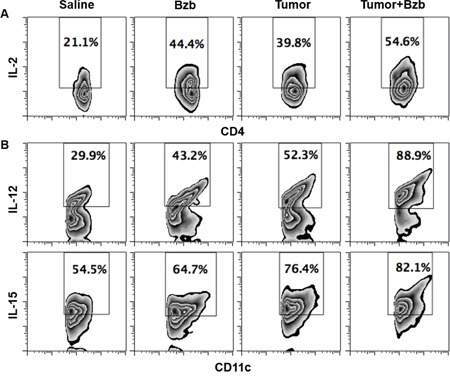
Bortezomib-mediated modulation of IL-2 secretion in CD4+T cells and IL-12 and IL-15 cytokines in CD11c+ cells of tumor-bearing mice 0.5 × 106 4T1HA tumor cells were injected orthotopically under mammary pads to establish tumors approximately 125 mm3 in size. Bzb (1 mg/kg body weight) was administered intravenously in mice. Spleens were harvested 4 h later for analysis. Intracellular secretion of IL-2 from gated CD4+T cells A. or IL-12 and IL-15 from CD11c+ cells B. is shown by flow cytometry. Numbers inside plots depict % positive cells. Representative data are shown from 2 independent experiments.

**Figure 7:**
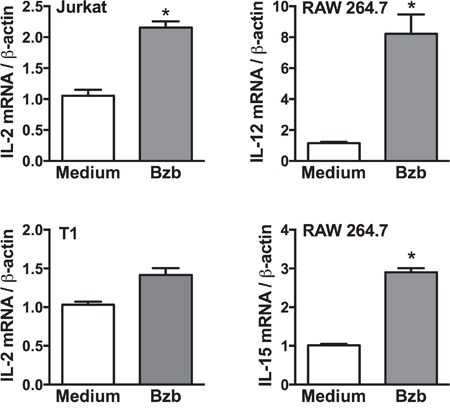
Cytokine expression in lymphoid and myeloid cell lines following bortezomib treatment Expression of target cytokine mRNA was analyzed by RT-PCR in cell lines: Jurkat (CD4+T cell), T1 (CD8+T cell), and RAW264.7 (macrophage) 4 h after 10 nM Bzb treatment. Data are presented as the ratio of target gene expression to housekeeping gene β-actin from two independent experiments. Values are means ± SD; *p <0.05; two-tailed t-test.

To determine the cellular source of cytokines modulated by bortezomib in tumor-bearing mice, we screened lymphoid and myeloid populations for the intracellular secretion of immunostimulatory cytokines. Although CD8^+^T cells or myeloid CD11b^+^ or CD11c^+^ cells did not show any noticeable increase in the intracellular production of IL-2 in the presence of bortezomib administration in 4T1HA tumor-bearing mice (data not shown), we observed a significant increase in the lymphoid CD4^+^T cells producing higher levels of IL-2 in bortezomib-treated mice (Figure 6A). Moreover, gated CD11c^high^ dendritic cells showed a remarkable increase in the production of intracellular IL-12 and IL-15 in bortezomib-treated mice (Figure 6B). The increase in these cytokines was not due to an increase in the frequency of CD4^+^T or CD11c^high^ cells, but was due to increase in the cytokine production per cell. We observed that bortezomib given at our tested therapeutic dose (1 mg/kg body weight) had no significant effects either on total leukocyte counts in control or tumor-draining LN, tumor-infiltrating lymphocytes, and spleen, or in subsets of CD4^+^T, CD8^+^T, and CD11c^+^ cells [33]. Further, following bortezomib treatment (10 nM; 4 h) *in vitro*, macrophage cell line RAW264.7 showed significant increases in both IL-12 and IL-15 mRNA expression and Jurkat (human CD4^+^T cell line), and T1 (human CD8^+^T cell line) showed an increase in IL-2 mRNA levels when compared with untreated cells (Figure 7). These results suggest that bortezomib administration can enhance adaptive immune mechanisms against tumor by increasing the secretion of IL-2, IL-12 and IL-15 from CD4^+^T helper cells and antigen-presenting dendritic cells, respectively.

### Bortezomib treatment increases cytokine receptor expression on tumor-infiltrating CD8^+^T lymphocytes

**Figure 8:**
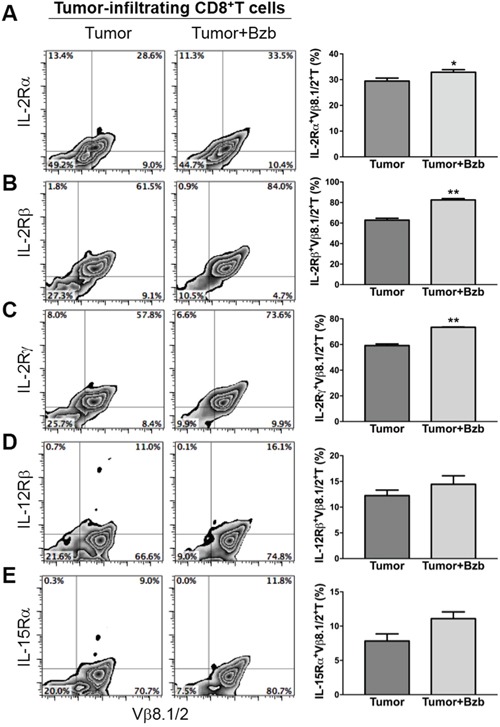
Bortezomib-mediated modulation of IL-2 receptor chains, IL-12Rβ, and IL-15Rα on tumor-infiltrating CD8+T cells Orthotopic tumors were established under mammary pads in Balb/c WT mice following injection of 2 × 106 4T1HA tumor cells. On day 14 (after the tumor reached an approximate size of 125 mm3), mice were injected intravenously with 1 mg/kg body weight of bortezomib. After 4 h, the mice were euthanized and single cell suspensions were obtained from the tumor mass harvested. The expression of cytokine receptors IL-2Rα **A.** IL-2Rβ **B.** IL-2Rγ **C.** IL-12Rβ **D.** and IL-15Rα **E.** is shown on tumor-infiltrating CD8+T cells by flow cytometry, with corresponding bar graphs presenting values as means ± SD. Numbers inside plots depict % positive cells. Representative data are shown from 2 independent experiments with 3 mice per group. *p<0.05, **p<0.01; two-tailed t-test.

Following our observation that bortezomib increases the secretion of immunostimulatory cytokines from helper T cells and antigen-presenting cells, we next wanted to determine if bortezomib treatment modulates the expression of these cytokine receptors on cytotoxic T cells at the site of tumor. We harvested the tumor mass from 4T1HA tumor-bearing mice and assessed the expression of cytokine receptors on tumor-infiltrating CD8^+^T cells. We observed that following bortezomib treatment, tumor-infiltrating HA_518-526_-specific CD8^+^T cells showed an enhanced expression of IL-2Rα, IL-2Rβ and IL-2Rγ compared to tumor-infiltrating CD8^+^T cells without bortezomib treatment (Figure 8A-8C). A trend of increased expression of IL-12β, and IL-15Rα was also observed on tumor-infiltrating CD8^+^T cells following bortezomib treatment compared to tumor-infiltrating CD8^+^T cells without bortezomib treatment (Figure 8D, 8E).

### Bortezomib augments PI3K/Akt/STAT5 signaling to enhance the expression of CD8^+^T cell effector molecules

**Figure 9:**
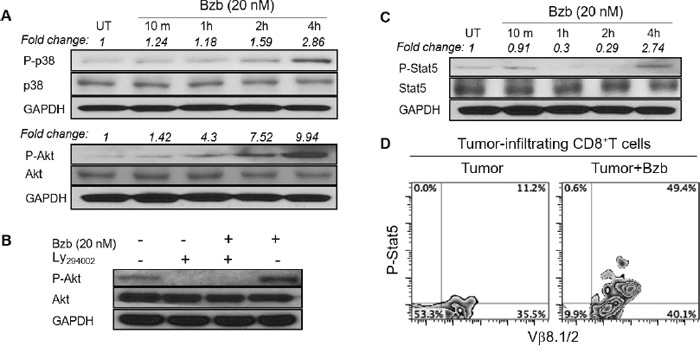
Bortezomib modulates the expression of phosphorylated p38, Akt, and STAT5 in CD8+T cells Levels of phosphorylated and total proteins were analyzed by Western blot in CD8+T cells following treatment in vitro with Bzb (20 nM) for the indicated duration of time. **A.** Protein levels of phosphorylated and total p38 and Akt. **B.** Protein levels of phosphorylated and total Akt following treatment with a combination of Bzb and PI3K inhibitor Ly294002 (50 μM). **C.** Protein levels of phosphorylated and total STAT5. The values on the Western blots represent fold change in expression analyzed by the densitometry with GAPDH used as a loading control. **D.** Orthotopic tumors were established in mammary pads of Balb/c WT mice following the injection of 2 × 106 4T1HA tumor cells. On day 14 (after the tumor reached an approximate size of 125 mm3), mice were injected intravenously with 1 mg/kg body weight of bortezomib. After 4 h, the mice were euthanized and single cell suspensions were obtained from the tumor mass harvested. Intracellular expression of phosphorylated STAT5 on tumor-infiltrating CD8+T cells was analyzed by flow cytometry. Numbers inside plots depict % positive cells. Representative data are from 2 individual experiments.

**Figure 10:**
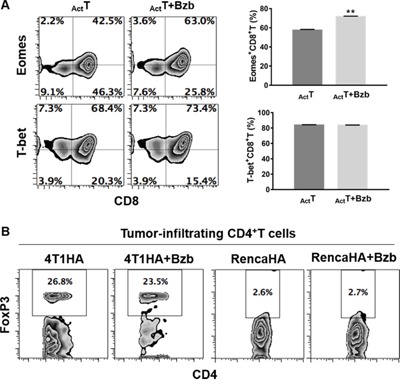
Effect of bortezomib treatment on the expression of transcription factors eomesodermin, T-bet and FoxP3 in T cells **A.** Purified CD8+T cells from spleens of naïve Balb/c mice were stimulated in vitro with soluble anti-mouse CD3 and CD28 antibodies (1 μg/ml each) for 48 h with or without treatment with Bzb (10 nM) for another 4 h. Intracellular expression of transcription factors Eomes and T-bet were analyzed by flow cytometry. Numbers inside plots depict % positive cells. Bar graphs present data as means ± SEM for Eomes+CD8+T cells and T-bet+CD8+T cells from 2 independent experiments. **p<0.01; two-tailed t-test. **B.** Orthotopic (4T1HA) or subcutaneous (RencaHA) tumors were established in Balb/c WT mice following the injection of 2 × 106 tumor cells. On day 14, mice were injected intravenously with 1 mg/kg body weight of bortezomib. After 4 h, the mice were euthanized and single cell suspensions were obtained from the tumor mass harvested. Intracellular expression of FoxP3 on tumor-infiltrating CD8+T cells was analyzed by flow cytometry. Numbers inside plots depict % positive cells. Representative data are from 2 individual experiments.

We assessed a panel of signaling molecules in CD8^+^T cells following bortezomib treatment. A time course of p38 and Akt phosphorylation following bortezomib (20 nM) treatment showed a time-dependent increase in their phosphorylation (Figure 9A). A 2-fold increase in p38 phosphorylation and a 7-fold increase in Akt phosphorylation was noted at 4 h post bortezomib treatment. We also observed that incubation of bortezomib-treated CD8^+^T cells with PI3K inhibitor, Ly294002, abrogated Akt phosphorylation (Figure 9B), suggesting that bortezomib-induced Akt phosphorylation was mediated *via* the PI3K pathway. We also screened bortezomib-treated CD8^+^T cells for a phospho-flow panel of all 6 members of STAT transcription factors. We observed a 3-fold increase in STAT5 phosphorylation in CD8^+^T cells at 4 h post bortezomib treatment (Figure 9C). Upregulation of STAT5 phosphorylation was also observed in Vβ8.1/2^+^CD8^+^T cells infiltrating orthotopic 4T1HA mammary tumors in mice treated with bortezomib (1 mg/kg body weight) (Figure 9D). No noticeable change was observed in the levels of other STAT members. We also analyzed the expression of T-box transcription factors eomesodermin and T-bet in CD8^+^T cells that were stimulated *in vitro* with soluble anti-mouse CD3 and CD28 antibodies (1 μg/ml each) for 48 h with or without treatment with bortezomib for another 4 h. We observed significant upregulation of intracellular eomesodermin but not T-bet expression in CD8^+^T cells (Figure 10A). CD4^+^T cells also did not show any change in T-bet expression (data not shown). Moreover, no change in the expression of FoxP3 transcription factor was observed in tumor-infiltrating CD4^+^T cells in 4T1HA and RencaHA tumor-bearing mice (Figure 10B). This suggests that bortezomib treatment upregulated signaling pathways leading to an increased expression of CD8^+^T cell effector molecules, without upregulating CD4^+^T regulatory immunosuppressive signaling.

**Figure 11:**
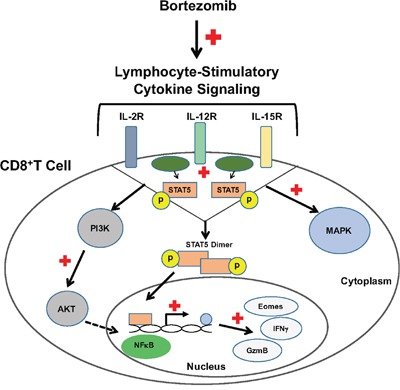
Bortezomib enhances cytokine receptor signaling to enhance the expression of CD8+T cell effector molecules Bortezomib treatment increases lymphocyte-stimulatory signaling through cytokines, IL-2, IL-12, and IL-15 via p38/PI3K/Akt/STAT5 pathways. This increases the transcription of effector molecules eomesodermin, granzyme-B and IFNγ in CD8+T lymphocytes.

Altogether, these results suggest the immunostimulatory potential of bortezomib to augment the strength of convergent signals from CD8^+^T cell signaling molecules such as TCR, IL-2, IL-12 and IL-15 cytokine receptors and downstream PI3K/Akt/STAT5 pathways to sustain the expression of CD8^+^T cell effector molecules in the tumor microenvironment as illustrated schematically in Figure 11.

## DISCUSSION

We report that in mice bearing kidney or breast adenocarcinomas that present a defined low-avidity epitope derived from hemagglutinin model antigen, bortezomib administration at 1 mg/kg body weight augments the expression of lymphocyte-stimulatory cytokines IL-2, IL-12, and IL-15 and their downstream signaling molecules. We observed that bortezomib treatment increased IL-2 production by CD4^+^T cells, IL-12 and IL-15 production from CD11c^+^ dendritic cells, and enhanced the expression of IL-2 receptor-α chain, IFNγ, granzyme-B, as well as the transcription factor eomesodermin on endogenous or adoptively transferred HA-specific CD8^+^T cells from bortezomib-treated tumor-bearing mice. We also noted increased phosphorylation of mitogen-activated protein kinase p38, Akt and transcription factor STAT5 in CD8^+^T cells following bortezomib treatment. These observations support the immunostimulatory potential of bortezomib with a capacity to sustain CD8^+^T cell effector function in the tumor microenvironment of solid tumors.

Tumor-mediated immunosuppression affects immune surveillance mechanisms and impedes the effectiveness of anti-cancer therapy [39]. The expression of immunoinhibitory molecules and cytokines, for example, IL-1β, VEGF, IL-10, and TGFβ and downregulation of immunostimulatory cytokines (e.g., IL-2, IL-12, IL-15) allow tumor to escape from immune control. IL-2 is a cytokine widely known to promote proliferation with a broad range of implications in tumor growth and control [40]. IL-2 promotes the expansion of T-regulatory cells expressing the ICOS molecule due to their increased expression of high-affinity IL-2 receptor CD25, which correlates with worse clinical outcome [41]. On the other hand, IL-15, another member of the IL-2 cytokine family, has more promising antitumor effects since it does not show overt side effects, such as vascular leak syndrome observed for IL-2 [42]. IL-15 promotes differentiation and proliferation of T and NK cells as well as induces long-lasting T cell memory by upregulating anti-apoptotic genes, such as *Bcl-xL* and *Bcl-2* [42]. Thus, IL-2 together with IL-15 sustains productive T cell proliferation, effector differentiation, and survival [43]. IL-12 is another immunostimulatory cytokine produced by primed antigen-presenting cells, and the consensus is that it supports CD8^+^T cell responses, Th1 lymphocyte differentiation, and NK cell function, all activities necessary for immune surveillance of tumors [44]. IL-12 by itself also has some antitumor activity mediated by its ability to induce IFNγ and elicit anti-angiogenic activity [44].

In our cancer models, we noted that the introduction of HA antigen into the tumor cells elicited some basal immune response. This is seen with increased IL-12 and IL-15 production in tumor-bearing mice as compared to control mice with no tumor. Treatment of tumor-bearing mice with bortezomib further increased the production of these lymphocyte-stimulatory cytokines IL-2, IL-12, and IL-15, which have significant impact on the development of a cytotoxic response mediated by CD8^+^T cells and NK cells. We also noted that IFNγ synthesis and control of metastatic growth are both dependent on IL-12 and IL-15 signaling since the blocking of these cytokines decreased IFNγ production and increased the number of tumor pulmonary nodules. Of note, bortezomib treatment restored IFNγ synthesis and inhibited metastasis in tumor-bearing mice even in the presence of neutralizing antibodies to IL-12 and IL-15. This indicates that bortezomib can activate alternative pathways leading to IFNγ production that could strengthen tumor immune surveillance. In particular, multiple cytokines such as IL-6, LIX (CXCL5), KC, etc. were upregulated and pro-angiogenic factor VEGF was downregulated following bortezomib treatment. Other studies also indicate that bortezomib inhibits the pro-tumor effects of IL-6, TGFβ, VEGF etc. and decreases tumor angiogenesis [15, 45].

Our data also show that bortezomib treatment increased phosphorylation of mitogen-activated protein kinase p38 and Akt as well as higher levels of phosphorylation of the transcription factor STAT5 in tumor-infiltrating CD8^+^T cells. Our recent studies showed that bortezomib can also increase the cytoplasmic and nuclear phosphorylation of NFκB p65 in CD8^+^T cells while maintaining IFNγ secretion to improve FasL-mediated tumor lysis [7, 33]. Recognition of antigen by the TCR activates the Ras-MAPK pathway, and costimulation provided by CD28 activates the phosphatidyl inositol-3-kinase pathway that synergizes to augment TCR-induced transcriptional regulation mediated by AP-1, NF-AT and NFκB [46]. In contrast to signaling by the TCR and CD28, the JAK/STAT pathways play a major role in signaling by cytokine receptors. The phenotype and functions of T cells can be modified by a wide array of cytokines, but IL-12 and/or IFN-α are distinguished by the fact that they can act as the ‘switch’ that determines whether a response to antigen will result in tolerance or full activation of antigen-specific CD8^+^T cells. Our results show predominant effects of bortezomib on both the p40 and p70 chains of IL-12 produced by dendritic cells. Signals provided by IL-12 and/or IFN-α/β are required for the activation of naïve CD8 T cells, and IL-2 is needed to sustain and further expand the effector cells if antigen persists [46]. The JAKs and STATs involved in the signaling pathways employed by these cytokines are potential targets for immunosuppression. Bortezomib treatment upregulated the expression of STAT5 in antigen-specific tumor-infiltrating CD8^+^T cells. STAT5 activation contributes to the stabilization of the gene expression program in CD8^+^T cells. Phosphorylation and nuclear translocation of STAT5 are triggered by IL-2 bound to its high-avidity receptor IL-2Rα (CD25), the expression of which is controlled by TCR stimulation; STAT5 is not activated in naive T cells even when cultured in the presence of IL-2 [47, 48]. This mechanism permits cytokine receptor signaling to rescue abortive TCR signaling, such as that induced in response to weak or partial TCR agonists by complementing it with the activation of STAT5 that binds to TNF receptor family costimulatory molecules and granzyme-B promoters to stabilize the CD8^+^T cell effector program. Furthermore, bortezomib-increased phosphorylation of Akt in T cells was abrogated by the PI3K inhibitor. The PI3K/Akt signaling pathway controls proliferation, differentiation, and survival in many cell types including T cells [49]. The cumulative strength of convergent signals from signaling molecules, such as TCR, costimulatory molecules, and cytokine receptors governs the magnitude of Akt activation, which in turn dictates the differentiation of cytotoxic T lymphocytes into terminal effectors or memory cells [50]. It is to be noted that in response to IL-15 augmentation upon bortezomib treatment, enhanced PI3K/Akt/STAT5 activation may support the maintenance and survival of effector and memory CD8^+^T cells as observed during viral infections [51].

Thus, bortezomib may act as a multifaceted immunomodulatory drug in addition to suppressing constitutive activation of NFκB in tumor cells, whereby it abrogates their resistance to cell death triggers [17, 28, 33, 52]. In another study, we have observed that bortezomib treatment in mice bearing various solid tumors resulted in an upregulated expression of Notch signaling components to facilitate interplay between Notch and NFκB signaling pathways in CD8^+^T lymphocytes [7]. Altogether, these data strongly support the case that bortezomib can augment the strength of activation signals in antigen-specific CD8^+^T cells in the tumor microenvironment *via* cytokine receptors and downstream PI3K/Akt/NFκB/STAT5 pathways, which together sustain the differentiation of CD8^+^T cells into terminal effectors.

It is worth noting that the concentration of bortezomib to a large extent determines the outcome of its effects on the immune system with higher concentrations in excess of 20 nM causing immunosuppressive effects while concentrations lower than 20 nM provide immunostimulatory effects [15, 22, 27, 32, 53–55]. Immunosuppressive effects of bortezomib were observed in *in vitro* studies where the doses used were high (>100 nM) [56, 57]; doses that cannot be extrapolated to the therapeutic situation *in vivo*. Only 10–20 nM concentrations of bortezomib are attainable *in vivo* [58]. For example, Blanco *et al* observed that bortezomib treatment at high concentrations (>100 nM) generated immunosuppressive CD4^+^CD25^+^ regulatory T cells to prevent graft versus host disease [59]. Whereas we observed an immunostimulatory effect in IL-2 secretion by CD4^+^T cells following low dose (~20 nM) bortezomib treatment that improved anti-tumor immunity. Therefore, therapeutic administration of a carefully optimized, minimal bortezomib dose customized for a disease condition would prove effective in providing immune benefits while limiting the negative immune effects. Considering possible mechanisms of bortezomib action based on available data, we propose that this proteasome inhibitor can affect tumor growth by changing the intrinsic activity of immune cells. It is noteworthy that chronic inflammation has an important impact in the development of tumors by generating a tumor-promoting microenvironment [60]. Bortezomib appears to release breaks on the immune system from the tumor-associated suppressive microenvironment, and thereby allow immune cells to become activated. Our recent studies showed that bortezomib can enhance FasL-mediated tumor lysis [33]. It is also known that bortezomib can make tumor cells sensitive to NK cell-mediated killing [29, 31, 61]. Moreover, bortezomib inhibited TGFβ-mediated suppression of IFNγ and granzyme-B expression in activated CD8^+^T cells [7]. These studies together with our results show that bortezomib can increase the levels of immunostimulatory cytokines IL-2, IL-12, and IL-15 and enhance their downstream signaling pathways to augment CD8^+^T cell effector function. Thus, bortezomib, being a reversible proteasome inhibitor with a short half-life, can be combined with other immunotherapies and may have major clinical implications to break tumor-induced tolerance in CD8^+^T cells and elicit a robust antitumor immune response in cancer patients with solid malignancies.

## MATERIALS AND METHODS

### Mice

Balb/c mice were purchased from Harlan (Indianapolis, IN) and mice were 6-8-week-old and 25-30 g at the time of use. TCRHA-transgenic Cln4 mice (*αβ* TCR specific for hemagglutinin HA_518–526_ peptide restricted to H-2K*^d^*) were provided by Linda A. Sherman (The Scripps Research Institute, La Jolla, CA), and were bred at Meharry Medical College (MMC) animal facility and genotyped according to the methods described earlier [35]. Mice were housed in filter-topped cages under specific pathogen-free conditions in MMC animal facilities. Mice were cared for in accordance with the procedures outlined in the National Institutes of Health *Guide for the Care and Use of Laboratory Animals* and Institutional Animal Care and Use Committee (IACUC) at MMC. MMC is accredited by the Association for Assessment and Accreditation of Laboratory Animal Care International and follows the Public Health Service Policy for the care and use of laboratory animals under pathogen-free conditions.

### Tumor cell lines

The murine mammary adenocarcinoma line 4T1.2HA (courtesy Suzanne Ostrand-Rosenberg, University of Maryland, Baltimore, MD), renal cell adenocarcinoma line RencaHA (courtesy Hyam I. Levitsky, John Hopkins University, Baltimore, MD) and fibrosarcoma line D459 (courtesy David Carbone, Ohio State University, Columbus, OH) were maintained in 10% FCS-supplemented standard RPMI-1640 (Gibco, Invitrogen) culture medium. Low-passage (< 5) tumor cell cultures were used for the experiments, and were regularly authenticated with reference stocks to ensure fidelity; routine sterility and Mycoplasma testing was performed regularly. Solid tumors were induced in syngeneic Balb/c WT by injecting 2 × 10^6^ 4T1HA or RencaHA cells orthotopically under mammary pads or subcutaneously, respectively, into the right flank for approximately 14 days. Following the establishment of palpable tumors of approximately 120 mm^3^ size, mice were injected with therapeutic dose of bortezomib (1 mg/kg body weight) intravenously, as we optimized previously [17], and roughly correlated to a transient 20 nM concentration on the basis of the observation that a mouse of 20-25 g weight has approximately a blood volume of about 1.5 ml. After 4 h lymphoid tissues were harvested for the preparation of single cell suspensions. The right inguinal and brachial LN were used as the tumor-draining LN, while inguinal and brachial LNs of the left side were used as contralateral LN.

### Cell harvesting and sample preparation

Tissues were harvested from mice after sedation with isoflourene and cervical dislocation. Single cells suspensions were made from tissues mashed/homogenized on the Falcon 40 μm cell strainers in petri dishes containing complete RPMI media. The media containing cells were transferred to labeled 15 mL conical tubes, then spun down at 1200 rpm for 5 min at 4°C. Cells were washed twice by aspirating media after spin, resuspending cell pellets in media, and centrifuging again. Splenocytes were suspended in 1 ml of ACK buffer (KD Medical, Columbia, MD) for 1 min at room temperature to lyse erythrocytes. The conical tube was filled with complete RPMI media following the 1 min treatment to neutralize the reaction and centrifuged. The pellet was resuspended in complete RPMI media. 10 μL of each sample were mixed with 10μL of trypan blue, placed on a slide, and cells were counted using a Countess (Invitrogen) machine for total cell counts and viability.

### Milliplex mouse cytokine/chemokine magnetic bead panel

Cell lysates were prepared from whole tissue or serum samples. Lysates and serum were both quantified by BCA Protein Assay Kit (Pierce), and 250 μg of protein was equalized and used per sample in the cytokine/chemokine magnetic bead panel plate (Millipore) as per the manufacturer's protocol. The plate was read on the MAGPIX instrument (Millipore) with xPONENT software. The median fluorescent intensity (MFI) data were saved and analyzed using a 5-parameter logistic or spline curve-fitting method for calculating cytokine/chemokines concentrations in samples.

### Antibodies and immunofluorescence surface and intracellular staining

RBC-depleted splenocytes and/or lymphocytes (1 × 10^6^) were plated in a 96-well U-bottom plate, then spun down at 1100 rpm for 5 min at 4°C. RPMI medium was flicked off and plate vortexed to break up the pellet. Appropriate dilutions of antibodies were prepared in flow buffer containing 0.5% FBS. 50μL of fluorochrome-labelled anti-mouse antibodies: CD4-PE, CD4-FITC, CD8-APC, CD8-PerCPCy5.5, Vβ8.1/2-PerCPcy5.5, CD11c-PerCPCy5.5, IL-2Rα-APC, IL-2Rβ-FITC, IL-2Rγ-PE, IL-12Rβ-PE and IL-15Rα-APC were added to the designated wells and gently shook. The 96-well plate was placed on ice to incubate for 30 min in the dark. After incubation, 100μL of flow buffer was added to each well and the plate was centrifuged at 1100 rpm for 5 min at 4°C for washing. Cells were washed again with 150μL. Cells were resuspended and fixed in 200μL of paraformaldehyde, covered, and placed in 4°C until acquired. All monoclonal antibodies were purchased from eBioscience and Biolegend.

Following the surface immunofluorescence staining, to measure intracellular protein levels, cells were treated with Cytofix/Cytoperm kit (BD Biosciences) according to the manufacturer's instructions. We used the following intracellular anti-mouse Abs: IL-2-FITC, IL-12-PE, IL-15-APC, IFNγ-PE, granzyme B-FITC, eomesodermin-PE, T-bet-APC, Foxp3-PE and a panel of anti-human/mouse phospho-STAT1-6-PE (eBiosciences and Biolegend). Intracellular staining was performed by the incubation of cells at appropriate dilutions in Permwash buffer for 30 min at 4°C in the dark.

For the cytokine neutralization experiment, anti-human/mouse IL-15 and anti-mouse IL-12 purified Abs as well as their isotype control anti-mouse IgG and anti-mouse IgG1, respectively, were purchased from Abcam.

### RNA isolation and quantitative PCR

Total RNA was extracted using an RNeasy mini kit (Qiagen) and quantitated by reading the optical density at 260 nm. Possible genomic DNA contamination was removed by on-column DNase digestion using the RNase-free DNase set. The cDNA was synthesized using iScript cDNA synthesis kit (Bio-Rad). Real-time quantitative RT-PCR (qRT-PCR) was performed using CFX-96 Real Time System (Bio-Rad). The iQ SYBR green supermix (Bio-Rad) and gene-specific PCR primers were used in a 20 μL reaction following protocols recommended by the manufacturer. The conditions used for the PCR were as follows: 95°C for 3 min (1 cycle), 94°C for 20 s, 55°C for 30 s, and 72°C for 40 s (40 cycles). Fold changes in mRNA expression were assessed by the ^ΔΔ^Ct method. Primer sequences are as follows: Murine IL-2 (forward: 5’-TGAGTCAGCAACTGTGGTGG-3’, reverse: 5’-GCCCTTGGGGCTTACAAAAAG-3’), murine IL-12 (forward: 5’-GGGACCAGGCCCTATTATGC-3’, reverse: 5’-GAC CAAAGCCAGCTCCTCAT-3’), murine IL-15 (forward: 5’-CGCCCAAAAGACTTGCAGTG-3’, reverse: 5’-GGTGGATTCTCTCTGAGCTGT-3’), murine β-actin (forward: 5’-AGTGTGACGTTGACATCCGTA-3’, reverse: 5’-GCCAGAGCAGTAATCTCCTTC-3’), human IL-2 (forward: 5’-AGAATC CCAAACTCACCAGGA-3’, reverse: 5’-TGTTTCAGTTCTGTGGCCTTC-3’), and human β-actin (forward: 5’-CTCGCCTTTGCCGATCC-3’, reverse: 5’-GGGGTACTTCAGGGTGAGGA-3’).

### Western blot analysis

Cell pellets from various tissues from tumor-bearing or naïve mice were lysed in complete lysis buffer including protease and phosphatase inhibitors. 50 μg of each protein sample was electrophoresed on NuPage 4-12% Bis-Tris gel (Novex Life Technologies) and transferred to polyvinylidenedifluoride membranes using an iBlot® Dry Blotting system (Life Technologies). The membrane was then blocked in 5% skimmed milk in 1X phosphate-buffered saline-Tween-20 (1X PBST) for 2 h at room temperature with gentle agitation. After blocking, the blots were incubated with specific primary antibodies for the phosphorylated and total levels of STAT5, Akt, and p38 in 1% BSA (in 1X PBST) overnight at 4°C with gentle agitation. After 5 washes of 5 min each in 1X PBST, blots were incubated with goat anti-rabbit horseradish peroxidase (Santa Cruz Biotechnology) at a dilution of 1:4000 in 1X PBST for 1.5 h, with agitation. The blots were rinsed again in 1X PBST, and developed by using chemiluminescence reagent (EMD Millipore, MA) and a Bio-Rad Image Station. The density of each protein band was determined by densitometric analysis using the imageJ software (NIH). Glyceraldehyde 3-phosphate dehydrogenase (GAPDH) levels were determined for each condition to verify that equal amounts of protein were loaded. In addition, the density of each protein band was normalized to GAPDH to determine relative protein expression to the internal control.

### Acquisition and data analysis

Flow samples were acquired on guava EasyCyte HT (Millipore) instrument with 50-200,000 cells acquired for each sample well. Gates for samples were determined by single color controls. Unstained and isotype controls were used to ascertain if any nonspecific binding of antibodies was present. All data were analyzed on FlowJo 7.6 software (TreeStar). Data were analyzed using either one-way ANOVA or two-tailed *t*-test using Graphpad Prism 7, with *p* ≤ 0.05 considered statistically significant.

## Abbreviations

HA: Hemagglutinin
4T1HA: 4T1.2 expressing HA
STAT: Signal transducer and activator of transcription, JAK, Janus kinase
TGFβ: Transforming growth factor beta
VEGF: Vascular endothelial growth factor
NFκB: Nuclear factor kappa-light-chain-enhancer of activated B cells
ICOS: Inducible T-cell costimulator
ACT: Adoptive cell immunotherapy
TIL: Tumor infiltrating lymphocytes
LN: Lymph node
RAG: Recombination activating gene
Eomes: Eomesodermin
MFI: Median fluorescent intensity
qRT-PCR: Real-time quantitative RT-PCR
